# Supplementary material for: Modeling glioblastoma heterogeneity as a dynamic network of cell states
Source: Mol Syst Biol. 2021 Sep 16;17(9):e10105. doi: 10.15252/msb.202010105 (PMC8444284; doi:10.15252/msb.202010105)
Supplement: Supplementary file 5 — Source Data for Figure 3 [file MSB-17-e10105-s001.zip › Figure3A_sourcedata/GSEA_3065/hallmarks_state2.GseaPreranked.1623416269049/HALLMARK_UV_RESPONSE_UP.html]

Details for gene set HALLMARK\_UV\_RESPONSE\_UP[GSEA]

|  || Dataset | state2 |
| Phenotype | NoPhenotypeAvailable |
| Upregulated in class | na\_pos |
| GeneSet | HALLMARK\_UV\_RESPONSE\_UP |
| Enrichment Score (ES) | 0.30506685 |
| Normalized Enrichment Score (NES) | 1.0278687 |
| Nominal p-value | 0.41073826 |
| FDR q-value | 0.49655408 |
| FWER p-Value | 1.0 |
Table: GSEA Results Summary

  

Fig 1: Enrichment plot: HALLMARK\_UV\_RESPONSE\_UP      
 Profile of the Running ES Score & Positions of GeneSet Members on the Rank Ordered List

  

| PROBE | GENE SYMBOL | GENE\_TITLE | RANK IN GENE LIST | RANK METRIC SCORE | RUNNING ES | CORE ENRICHMENT || 1 | FEN1 |  |  | 107 | 0.371 | 0.0236 | Yes |
| 2 | RFC4 |  |  | 126 | 0.339 | 0.0538 | Yes |
| 3 | BTG3 |  |  | 173 | 0.295 | 0.0768 | Yes |
| 4 | PSMC3 |  |  | 275 | 0.242 | 0.0888 | Yes |
| 5 | LHX2 |  |  | 329 | 0.223 | 0.1043 | Yes |
| 6 | POLE3 |  |  | 332 | 0.223 | 0.1251 | Yes |
| 7 | CDK2 |  |  | 382 | 0.208 | 0.1396 | Yes |
| 8 | AP2S1 |  |  | 385 | 0.208 | 0.1591 | Yes |
| 9 | PPAT |  |  | 401 | 0.200 | 0.1764 | Yes |
| 10 | PDAP1 |  |  | 454 | 0.190 | 0.1888 | Yes |
| 11 | DNAJA1 |  |  | 513 | 0.178 | 0.1994 | Yes |
| 12 | PPIF |  |  | 580 | 0.166 | 0.2080 | Yes |
| 13 | SELENOW |  |  | 583 | 0.166 | 0.2235 | Yes |
| 14 | EIF5 |  |  | 699 | 0.150 | 0.2254 | Yes |
| 15 | POLR2H |  |  | 787 | 0.140 | 0.2293 | Yes |
| 16 | STIP1 |  |  | 794 | 0.139 | 0.2417 | Yes |
| 17 | CEBPG |  |  | 860 | 0.131 | 0.2472 | Yes |
| 18 | PARP2 |  |  | 886 | 0.129 | 0.2568 | Yes |
| 19 | CCNE1 |  |  | 888 | 0.129 | 0.2689 | Yes |
| 20 | FKBP4 |  |  | 1079 | 0.113 | 0.2592 | Yes |
| 21 | CCND3 |  |  | 1164 | 0.107 | 0.2603 | Yes |
| 22 | MRPL23 |  |  | 1176 | 0.106 | 0.2691 | Yes |
| 23 | DNAJB1 |  |  | 1227 | 0.103 | 0.2735 | Yes |
| 24 | OLFM1 |  |  | 1263 | 0.101 | 0.2793 | Yes |
| 25 | CDC5L |  |  | 1337 | 0.096 | 0.2805 | Yes |
| 26 | HNRNPU |  |  | 1369 | 0.094 | 0.2860 | Yes |
| 27 | KLHDC3 |  |  | 1397 | 0.092 | 0.2919 | Yes |
| 28 | ATP6V1F |  |  | 1461 | 0.089 | 0.2935 | Yes |
| 29 | DDX21 |  |  | 1486 | 0.087 | 0.2992 | Yes |
| 30 | BID |  |  | 1508 | 0.086 | 0.3051 | Yes |
| 31 | GRPEL1 |  |  | 1664 | 0.079 | 0.2959 | No |
| 32 | PDLIM3 |  |  | 1841 | 0.071 | 0.2837 | No |
| 33 | CYB5B |  |  | 1850 | 0.070 | 0.2895 | No |
| 34 | CDC34 |  |  | 1959 | 0.066 | 0.2842 | No |
| 35 | CASP3 |  |  | 2028 | 0.064 | 0.2829 | No |
| 36 | CA2 |  |  | 2111 | 0.061 | 0.2798 | No |
| 37 | GGH |  |  | 2149 | 0.059 | 0.2815 | No |
| 38 | MARK2 |  |  | 2162 | 0.059 | 0.2858 | No |
| 39 | ARRB2 |  |  | 2174 | 0.058 | 0.2901 | No |
| 40 | NUP58 |  |  | 2430 | 0.050 | 0.2675 | No |
| 41 | CLTB |  |  | 2455 | 0.049 | 0.2695 | No |
| 42 | SHOX2 |  |  | 2461 | 0.049 | 0.2736 | No |
| 43 | MSX1 |  |  | 2551 | 0.046 | 0.2684 | No |
| 44 | PRKACA |  |  | 2554 | 0.046 | 0.2726 | No |
| 45 | SIGMAR1 |  |  | 2594 | 0.045 | 0.2726 | No |
| 46 | RHOB |  |  | 2690 | 0.042 | 0.2664 | No |
| 47 | ALAS1 |  |  | 2766 | 0.040 | 0.2621 | No |
| 48 | E2F5 |  |  | 2932 | 0.035 | 0.2478 | No |
| 49 | ENO2 |  |  | 3006 | 0.034 | 0.2432 | No |
| 50 | CNP |  |  | 3251 | 0.028 | 0.2197 | No |
| 51 | CHRNA5 |  |  | 3301 | 0.028 | 0.2171 | No |
| 52 | SOD2 |  |  | 3366 | 0.026 | 0.2127 | No |
| 53 | TGFBRAP1 |  |  | 3430 | 0.025 | 0.2083 | No |
| 54 | SLC25A4 |  |  | 3452 | 0.025 | 0.2084 | No |
| 55 | WIZ |  |  | 3468 | 0.024 | 0.2091 | No |
| 56 | STK25 |  |  | 3490 | 0.024 | 0.2092 | No |
| 57 | AMD1 |  |  | 3505 | 0.024 | 0.2099 | No |
| 58 | SPR |  |  | 3581 | 0.022 | 0.2040 | No |
| 59 | PRPF3 |  |  | 3651 | 0.021 | 0.1986 | No |
| 60 | JUNB |  |  | 3923 | 0.017 | 0.1711 | No |
| 61 | AGO2 |  |  | 4202 | 0.012 | 0.1425 | No |
| 62 | ALDOA |  |  | 4435 | 0.008 | 0.1184 | No |
| 63 | CDO1 |  |  | 4513 | 0.007 | 0.1108 | No |
| 64 | TST |  |  | 4725 | 0.004 | 0.0885 | No |
| 65 | YKT6 |  |  | 4828 | 0.002 | 0.0778 | No |
| 66 | BAK1 |  |  | 4847 | 0.002 | 0.0761 | No |
| 67 | NXF1 |  |  | 5224 | -0.004 | 0.0361 | No |
| 68 | MGAT1 |  |  | 5429 | -0.007 | 0.0149 | No |
| 69 | BCL2L11 |  |  | 5510 | -0.009 | 0.0072 | No |
| 70 | ASNS |  |  | 5539 | -0.009 | 0.0050 | No |
| 71 | ATP6V1C1 |  |  | 5554 | -0.009 | 0.0044 | No |
| 72 | RXRB |  |  | 5748 | -0.012 | -0.0152 | No |
| 73 | STARD3 |  |  | 5787 | -0.013 | -0.0180 | No |
| 74 | UROD |  |  | 5835 | -0.014 | -0.0218 | No |
| 75 | CHKA |  |  | 6122 | -0.019 | -0.0506 | No |
| 76 | ACAA1 |  |  | 6129 | -0.019 | -0.0495 | No |
| 77 | POLG2 |  |  | 6142 | -0.019 | -0.0490 | No |
| 78 | NFKBIA |  |  | 6199 | -0.020 | -0.0531 | No |
| 79 | PTPRD |  |  | 6440 | -0.024 | -0.0766 | No |
| 80 | TYRO3 |  |  | 6479 | -0.025 | -0.0783 | No |
| 81 | FURIN |  |  | 6544 | -0.026 | -0.0827 | No |
| 82 | EPHX1 |  |  | 6547 | -0.026 | -0.0804 | No |
| 83 | ATF3 |  |  | 6737 | -0.030 | -0.0978 | No |
| 84 | KCNH2 |  |  | 6934 | -0.035 | -0.1155 | No |
| 85 | EIF2S3 |  |  | 7040 | -0.038 | -0.1232 | No |
| 86 | DGAT1 |  |  | 7121 | -0.040 | -0.1281 | No |
| 87 | PPP1R2 |  |  | 7206 | -0.042 | -0.1331 | No |
| 88 | FOS |  |  | 7222 | -0.043 | -0.1307 | No |
| 89 | CLCN2 |  |  | 7269 | -0.044 | -0.1314 | No |
| 90 | CDKN1C |  |  | 7524 | -0.051 | -0.1538 | No |
| 91 | IRF1 |  |  | 7563 | -0.052 | -0.1530 | No |
| 92 | GRINA |  |  | 7750 | -0.058 | -0.1674 | No |
| 93 | RPN1 |  |  | 7869 | -0.062 | -0.1742 | No |
| 94 | NPTXR |  |  | 7889 | -0.063 | -0.1703 | No |
| 95 | TAP1 |  |  | 7963 | -0.066 | -0.1719 | No |
| 96 | SLC6A8 |  |  | 8016 | -0.068 | -0.1711 | No |
| 97 | CYB5R1 |  |  | 8101 | -0.071 | -0.1733 | No |
| 98 | BTG1 |  |  | 8156 | -0.075 | -0.1720 | No |
| 99 | PPT1 |  |  | 8369 | -0.087 | -0.1865 | No |
| 100 | HSPA13 |  |  | 8415 | -0.090 | -0.1829 | No |
| 101 | TFRC |  |  | 8650 | -0.107 | -0.1978 | No |
| 102 | SQSTM1 |  |  | 8790 | -0.121 | -0.2013 | No |
| 103 | GLS |  |  | 8889 | -0.134 | -0.1991 | No |
| 104 | NTRK3 |  |  | 8986 | -0.146 | -0.1956 | No |
| 105 | HMOX1 |  |  | 9084 | -0.163 | -0.1906 | No |
| 106 | IL6ST |  |  | 9090 | -0.164 | -0.1756 | No |
| 107 | HYAL2 |  |  | 9174 | -0.182 | -0.1673 | No |
| 108 | BTG2 |  |  | 9239 | -0.207 | -0.1546 | No |
| 109 | IGFBP2 |  |  | 9240 | -0.208 | -0.1349 | No |
| 110 | TMBIM6 |  |  | 9264 | -0.219 | -0.1167 | No |
| 111 | BSG |  |  | 9331 | -0.253 | -0.0999 | No |
| 112 | CCK |  |  | 9433 | -0.576 | -0.0562 | No |
| 113 | CTSV |  |  | 9434 | -0.601 | 0.0006 | No |
Table: GSEA details [plain text format]

  

Fig 2: HALLMARK\_UV\_RESPONSE\_UP: Random ES distribution      
 Gene set null distribution of ES for **HALLMARK\_UV\_RESPONSE\_UP**
